# Associations of Subjective Memory Concerns with Chronic Stress and Social Support in Mexican Americans: A Longitudinal Study

**DOI:** 10.1002/alz.088455

**Published:** 2025-01-03

**Authors:** Alexa S. Gonzalez, Olivia E. Atherton, Christopher A. Krause, Joshua M. Garcia, Michelle N. Martinez, Luis D. Medina

**Affiliations:** ^1^ University of Houston, Houston, TX USA

## Abstract

**Background:**

The progression of Alzheimer’s disease and related dementias (ADRDs) from prodromal state to dementia syndrome prompts researchers to identify early markers of cognitive decline. One potential risk marker is subjective memory concerns (SMCs). Individuals with greater perceived stress often report more cognitive concerns. Moreover, increased social support is linked to lower perceived stressors. Our previous cross‐sectional work with an ethnoracially diverse sample of older adults reveals a positive relationship between chronic stress and SMCs, adjusting for demographic factors. It remains unknown if chronic stress and social support are longitudinally associated with SMCs (at the between‐ and within‐person levels), particularly among older Hispanic/Latin Americans, who are notably underrepresented in ADRD research. The present research aims to address these gaps.

**Method:**

Data for 84 Mexican Americans (Age = 62±6.97; Education = 10±3.57) were collected across three waves (24‐to‐30‐month intervals) via the Health and Aging Brain Study: Health Disparities (HABS‐HD). Exclusion criteria included depression, stroke, Alzheimer’s disease, and/or other dementia diagnoses. Participants self‐reported subjective memory concerns, chronic stress, and social support levels. Multilevel models were conducted to explore longitudinal associations between chronic stress and social support on SMCs, controlling for age, gender, and education.

**Results:**

Findings showed significant between‐subject effects of chronic stress (β = .15, *p* = .018, 95% CI [.03, .27]) and of social support (β = ‐.26, *p* < .001, 95% CI [‐.40, ‐.12]) on SMCs. There were no significant within‐person effects of chronic stress (β = .046, *p* = .122, 95% CI [‐.012, .10]) or social support (β = ‐.045, *p* = .098, 95% CI [‐.11, .0093]) on SMCs.

**Conclusions:**

Individuals with higher chronic stress and lower social support tended to report more memory concerns compared to individuals with lower chronic stress and higher social support. These findings suggest that Mexican Americans facing stressors or having fewer social supports may be a group particularly at risk for ADRDs. Future research may examine if social support mediates the between‐person effects of long‐term stressors on SMCs through a longitudinal mediation model.

